# Clinical and genomic characteristics of metabolic syndrome in colorectal cancer

**DOI:** 10.18632/aging.202474

**Published:** 2021-02-11

**Authors:** Yanyan Li, Jungang Zhao, Xiaoli Wu, Yini Zhang, Yin Jin, Weiyang Cai

**Affiliations:** 1 Department of Ultrasound, The Second Affiliated Hospital and Yuying Children’s Hospital of Wenzhou Medical University, Wenzhou, Zhejiang Province, China; 2 Department of Hepatobiliary Surgery, The First Affiliated Hospital of Wenzhou Medical University, Wenzhou, China; 3 Department of Gastroenterology, The First Affiliated Hospital of Wenzhou Medical University, Wenzhou, China

**Keywords:** metabolic syndrome (MetS), colorectal cancer, multi-omics, drug sensitivity

## Abstract

Metabolic syndrome (MetS) is characterized by a group of metabolic disturbances which leads to the enhanced risk of cancer development. Elucidating the mechanisms between these two pathologies is essential to identify the potential therapeutic molecular targets for colorectal cancer (CRC). 716 colorectal patients from the First and Second Affiliated Hospital of Wenzhou Medical University were involved in our study and metabolic disorders were proven to increase the risk of CRC. The prognostic value of the MetS factors was analyzed using the Cox regression model and a clinical MetS-based nomogram was established. Then by using multi-omics techniques, the distinct molecular mechanism of MetS genes in CRC was firstly systematically characterized. Strikingly, MetS genes were found to be highly correlated with the effectiveness of targeted chemotherapy administration, especially for mTOR and VEGFR pathways. Our results further demonstrated that overexpression of MetS core gene IL6 would promote the malignancy of CRC, which was highly dependent on mTOR-S6K signaling. In conclusion, we comprehensively explored the clinical value and molecular mechanism of MetS in the progression of CRC, which may serve as a candidate option for cancer management and therapy in the future.

## INTRODUCTION

Colorectal cancer (CRC) is one of the most fatal cancers, in both men and women [1]. More than 1.8 million people worldwide were diagnosed with CRC in 2018, which was estimated by Global Cancer Incidence, Mortality, and Prevalence (GLOBOCAN) to account for 10.2% of all cancers [2]. There are many significant risk factors associated with the development of CRC, such as inflammatory bowel disease (IBD) (including ulcerative colitis and Crohn's disease), cancer history in a first-degree relative, obesity, smoking, irregular diet and the use of some drugs (e.g., nonsteroidal anti-inflammatory drugs and postmenopausal hormone replacement).

Metabolic syndrome (MetS) is a disease that includes at least three of the following five items: high blood pressure, high blood sugar, high triglycerides (TG), reduced low-density lipoprotein cholesterol (HDL-C) and high body mass index (BMI) [3]. The incidence of MetS is increasing dramatically worldwide, although the global data is hard to measure. However, as we know, the prevalence of diabetes has reached 8.8% worldwide and MetS is 3 times more than diabetes, from which we can speculate that over a billion people may be affected with MetS in the world currently [4]. MetS and its components have been proven increase the risk of various tumors, such as colorectal cancer, breast cancer and kidney cancer, and significantly augmented corresponding mortality [5]. Previous studies have demonstrated that MetS also increased the risk of postoperative death of colorectal cancer by 2.98 times [5]. Diabetes mellitus (DM) was reported to be a possible prognostic factor for progression free survival (PFS) in localized CRC [6], and modulation of BMI might reduce the risk of CRC mortality [7]. There are many hypotheses contribute to this phenomenon. Traditionally, adipose tissues were considered a place storing lipids. However, lipid metabolism disorder has recently been proven to function in modulating various signaling cascades and integrating systemic metabolism [8]. Moreover, insulin resistance could lead to hypertriglyceridemia through the synthesis of non-esterified fatty acids and triglycerides as well as the accumulation of fat tissue, which was involved in the development of colorectal carcinoma [9]. More seriously, the increasing of MetS gave rise to drug dysregulation and added the probability of chemotherapy side-effects. Thus, it is urgent to explore the specific mechanism underlying CRC metabolism to further predict and administer individual therapy.

The main purpose of our study was to identify the relationship between MetS and the progression of CRC. A MetS-related model was established to predict the prognosis of CRC and a nomogram was built for better application and use in clinical settings. Further study found that MetS-genes were associated with the effectiveness of targeted chemotherapy for CRC and the MetS core gene IL6 would promote the malignancy of colorectal cancer through mTOR-S6K signaling.

## RESULTS

### Association of metabolic syndrome with clinicopathological features

At first, a total of 716 CRC patients from Wenzhou Medical University met the inclusion criteria from 2014 January 1st to 2016 January 1st. As of March of 2020, 211 patients died during follow-up, none lost follow-up. About 49.4% of the patients only received surgery, while the others received both adjuvant postoperative therapy and surgery. Baseline clinicopathological parameters were summarized in Supplementary Table 1.

Metabolic syndrome is conferred as central obesity, dyslipidemia, hyperglycemia, insulin resistance, and hypertension. Metabolic disorders were proven to involve in increased tumor risk. In our study, the proportions of patients with overweight, hypertension, diabetes, and dyslipidemia were 27.5%, 12.2% and 59.5%, respectively. There were 108 patients diagnosed with metabolic syndrome (meeting over three requirements) and 608 patients failed to meet the diagnostic criteria. Correlation values between metabolic syndrome indexes and clinicopathological features were shown in Table 1.

**Table 1:** Clinicopathological characteristics of 716 colorectal cancer patients grouped by BMI, hypertension, diabetes, dyslipidemia and metabolic syndrome.

**Characteristics**	**BMI**			**DG**		**HP**		**HDL**		**TG**		**MetS**			
	**<18.5**	**18.5-25**	**≥25**	**No**	**Yes**	**No**	**Yes**	**<0.9**	**≥0.9**	**<1.7**	**≥1.7**	**0**	**1**	**2**	**3-5**
Sex			**0.026**		0.250		0.430		0.382		0.007				0.100
Male	34	296	95	370	55	304	121	202	223	295	131	98	157	105	64
Female	39	197	50	258	29	213	74	146	141	225	62	89	88	67	42
T stage			0.174		**<0.001**		**<0.001**		**<0.001**		**<0.001**				0.872
T1	4	28	2	32	2	25	9	15	19	22	12	11	9	10	4
T2	6	43	19	60	8	52	16	30	38	44	24	18	24	16	10
T3	12	52	19	72	11	57	26	38	45	57	26	16	31	24	12
T4	53	371	107	466	65	385	146	267	264	399	132	142	183	124	82
N stage			0.068		0.255		**0.079**		0.539		0.482				0.085
N0	32	271	66	329	40	279	90	181	188	264	105	106	116	98	49
N1	22	112	47	161	20	130	51	83	98	131	50	48	71	35	27
N2	21	111	34	140	26	110	56	86	80	127	39	33	60	41	32
M stage			**0.014**		**<0.001**		**0.011**		0.868		**0.039**				0.409
M0	54	415	128	531	66	444	153	291	306	425	172	157	212	147	85
M1	21	79	19	99	20	75	44	59	60	97	22	30	35	27	23
Stage			**<0.001**		**<0.001**		**<0.001**		**<0.001**		**<0.001**				0.332
1	7	61	19	75	12	61	26	42	45	62	25	24	25	24	14
2	20	195	44	233	26	202	57	124	135	186	73	77	84	66	32
3	27	160	65	224	28	182	70	126	126	178	74	56	101	55	40
4	21	183	19	98	20	74	44	58	60	96	22	30	37	29	22
CEA			**0.014**		**0.009**		0.162		0.440		**0.008**				0.512
<5	45	302	79	387	40	317	109	213	213	326	100	117	146	105	58
≥5	30	191	68	244	46	202	88	136	153	196	94	70	101	69	50
Renal failure			0.498		**<0.001**		**<0.001**		**<0.001**		0.881				**<0.001**
No	59	414	120	535	58	462	131	280	313	433	160	164	221	138	70
Yes	16	80	27	95	28	57	66	70	53	89	34	23	26	36	36
Chemotherapy			**0.002**		0.883		**0.022**		**0.002**		0.842				0.068
No	43	253	65	317	44	248	113	196	165	262	99	81	124	93	63
Yes	32	163	82	313	42	271	84	154	201	260	95	106	123	81	45

### Impact of metabolic syndrome on OS in patients with colorectal cancer

**Figure 1:**
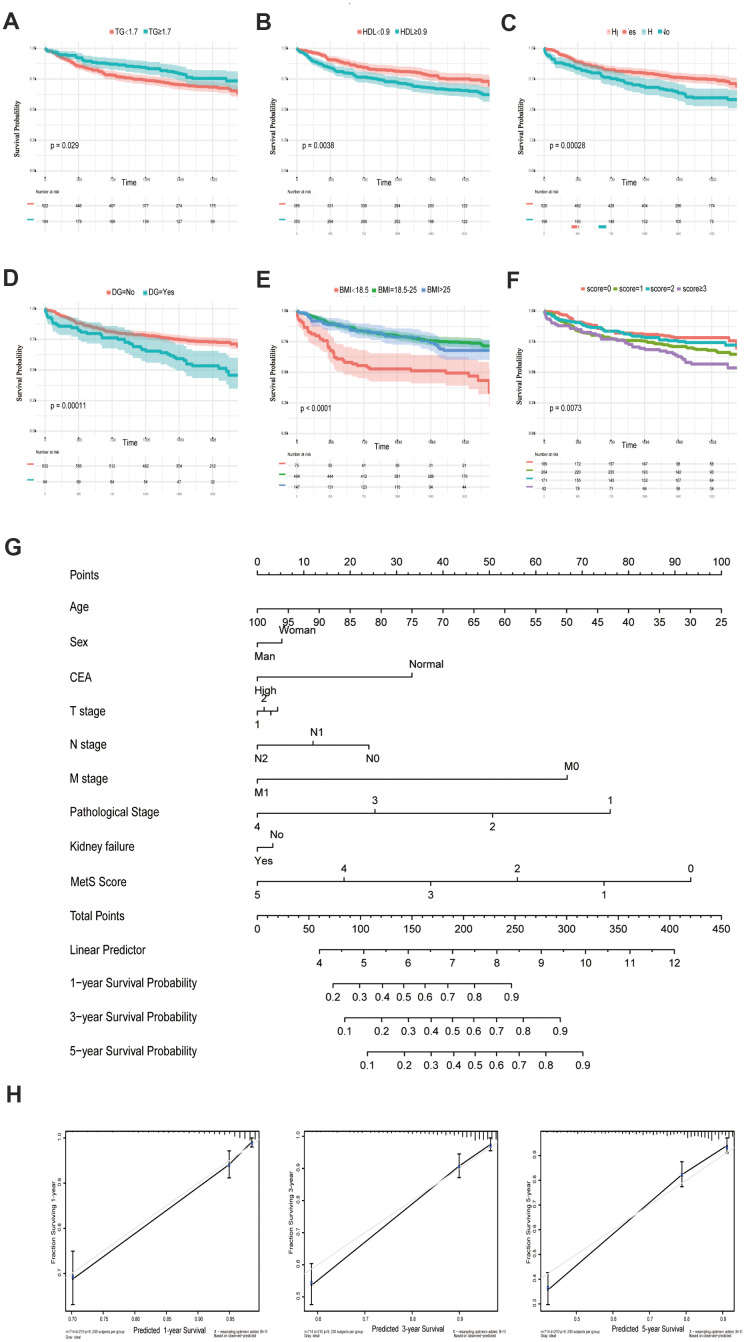
**Kaplan-Meier curves for CRC patients stratified by metabolic syndrome.** Kaplan-Meier analysis of overall survival (OS) of TG (**A**), HDL (**B**), hypertension (**C**), diabetes mellitus (**D**), BMI (**E**) and metabolic syndrome (**F**); (**G**). Nomogram developed by integrating metabolic syndrome and clinical pathological parameters for predicting 1-, 3-, 5-year survival of CRC patients; (**H**). Calibration curve for risk of 1-, 3-, 5-year survival of CRC metabolic syndrome.

The following metabolic variables had significant differences in survival rates within their respective groups by using univariate analysis. There were statistically significant differences between the groups with respect to TG, HDL, HP, DG and BMI (each parameter with P< 0.05, Figure 1A–1E, Table 2). The univariate analysis demonstrated that compared with normal blood glucose level patients, CRC patients with diabetes showed a poor prognosis in OS (P = 0.00011). Low BMI can be considered as a predictor of poor survival in OS. In particular, the underweight group has the worst prognosis, while the normal and overweight groups have similar prognosis (P<0.0001). In the same way, an increasing in HDL could lead to the increase in the mortality of CRC (P = 0.0038). Overall, patients diagnosed with metabolic syndrome (score ≥ 3) had the worst survival outcome among the enrolled population (Figure 1F). The multivariate analysis showed that only BMI (P < 0.001), diabetes (P = 0.046), TG (P = 0.001), and metabolic syndrome (P < 0.001) were significantly independently associated with OS.

**Table 2:** Univariate and multivariate Cox hazards analysis for OS in 716 patients with colorectal cancer.

**Variable**	**Univariate analysis**		**Multivariate analysis**	**P**
	**HR (95%CI)**	**P**	**HR (95%CI)**	
**Age**		**<0.001**		**0.01**
<60	Reference			
>60	0.591(0.440-0.795)		1.034(1.011-1.060)	
**Gender**		0.086		0.758
Male				
Female	1.279(0.966-1..695)		1.087(0.639-1.849)	
**CEA**		0.125		0.252
<				
>	1.156(0.829-1.548)		1.210(0.726-1.650)	
**T stage**		**<0.001**		**0.028**
T1				
T2	2.264(1.087-6.422)	0.032	1.228(0.342-4.407)	0.753
T3	1.201(0.437-3.305)	0.722	0.836(0.217-3.223)	0.795
T4	0.574(0.175-1.880)	0.359	0.583(0.176-1.929)	0.376
**N stage**		**<0.001**		**0.039**
N0				
N1	5.536(4.001-7.632)	<0.001	0.880(0.499-1.552)	0.659
N2	1.862(1.293-2.737)	0.001	1.496(0.884-2.440)	0.138
**M stage**		**<0.0001**		0.730
M0				
M1	8.236(6.321-10.885)		0.771(0.176-3.374)	
**Stage**		**<0.001**		**0.001**
1				
2	12.321(7.971-15.325)	<0.001	10.362(1.599-60.164)	0.014
3	3.162(1.584-6.314)	0.01	1.782(0.504-6.306)	0.370
4	1.191(0.596-2.496)	0.643	0.779(0.227-2.672)	0.691
**BMI**		**<0.001**		**<0.001**
<18.5				
18.5-25	0.414(0.270-0.636)	<0.001	2.427(1.554-3.791)	<0.001
>25	0.355(0.250-0.505)	<0.001	0.826(0.578-1.180)	0.293
**Hypertension**		**<0.001**		0.821
No				
Yes	1.702(1.288-2.249)		1.036(0.761-1.412)	
**Diabetes**		**0.0008**		**0.0456**
No				
Yes	1.935(1.367-2.722)		1.430(1.097-2.057)	
**HDL**		**0.03**		0.125
<0.9				
>0.9	0.679(0.503-0.966)		0.765(0.544-1.077)	
**TG**		**0.004**		**0.001**
<1.7				
>1.7	1.491(1.135-1.959)		1.659(1.247-2.209)	
**Kidney**		0.669		0.156
No				
Yes	1.045(0.853-1.281)		1.291(0.907-1.837)	
**MetS**		**<0.001**		**<0.001**

An OS nomogram was constructed to predict 1-, 3- and 5-year overall survival of colorectal cancer (Figure 1G). Total scores were summations of each variable based on the intersection of the vertical line. As shown in Figure 1G, metabolic syndrome and age contributed the most risk points (ranged, 0-100), whereas the other clinical information contributed much less (ranged, 0-75). By using this nomogram, we could convert each clinical index to the corresponding point, and then calculated the total point, which was used to evaluate the 1-year, 3-year, and 5-year survival rate. Moreover, decision curve analysis showed the high accuracy of the predictive prognostic of MetS score for 1-, 3- and 5-year OS possibility (Figure 1H).

### Summary and analysis of the genomic mechanism of metabolic syndrome

**Figure 2:**
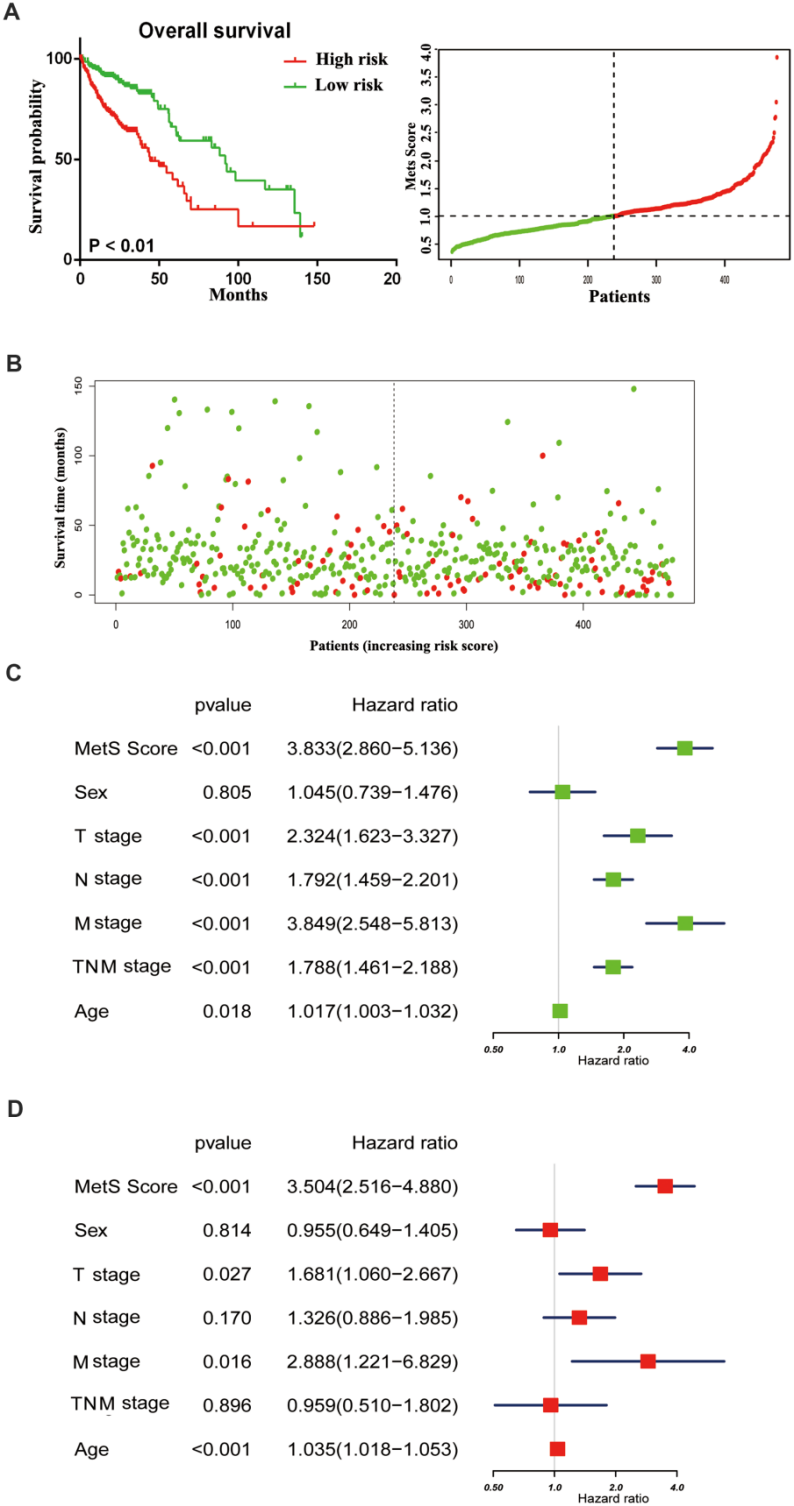
**The distribution of gene MetS Score in the TCGA cohort.** (**A**). K-M survival curve of the low- and high- MetS Score for TCGA CRC patients; (**B**). The distributions of the MetS Score and survival status for each CRC patients; (**C**). Forest plot summary the univariable analyses of overall survival of gene MetS Score; (**D**). Forest plot summary the multivariable analyses of overall survival of gene MetS Score.

With the rapid development of oncotherapy, metabolism regulation, as a promising predictor, has received more and more attention. Aforementioned epidemiological data have proven the tight connection between metabolic syndrome and increased cancer risk. Nevertheless, the mechanisms that link metabolic disorders and cancer risk remain unknown. The possible association mechanism between these two risk factors will be firstly described in this paper, focusing on the shedding light on candidate signature genes and biological events occurring in CRC progression. We identified 11 key metabolic genes, including TGFB, IGF2BP1, IGFL1, IGF2BP3, CHS2, IGF1R, IL6, IL6R, CSF1, CSF3 and IGF2 from recognized literature [5, 10]. Subsequently, we constructed a gene metabolic score via Cox regression analyses. As shown in Figure 2A, 2B, CRC patients with high metabolic signature had significantly poorer OS than patients with low metabolic signature. Next, univariate and multivariate Cox regression analyses were constructed to evaluate the prognostic significance of the gene metabolic signature combined with various clinicopathologic factors (Figure 2C, 2D). Gene MetS score was also proven to be an independent prognostic factor and had strong predictive power.

### Identifying metabolic -related modules

**Figure 3:**
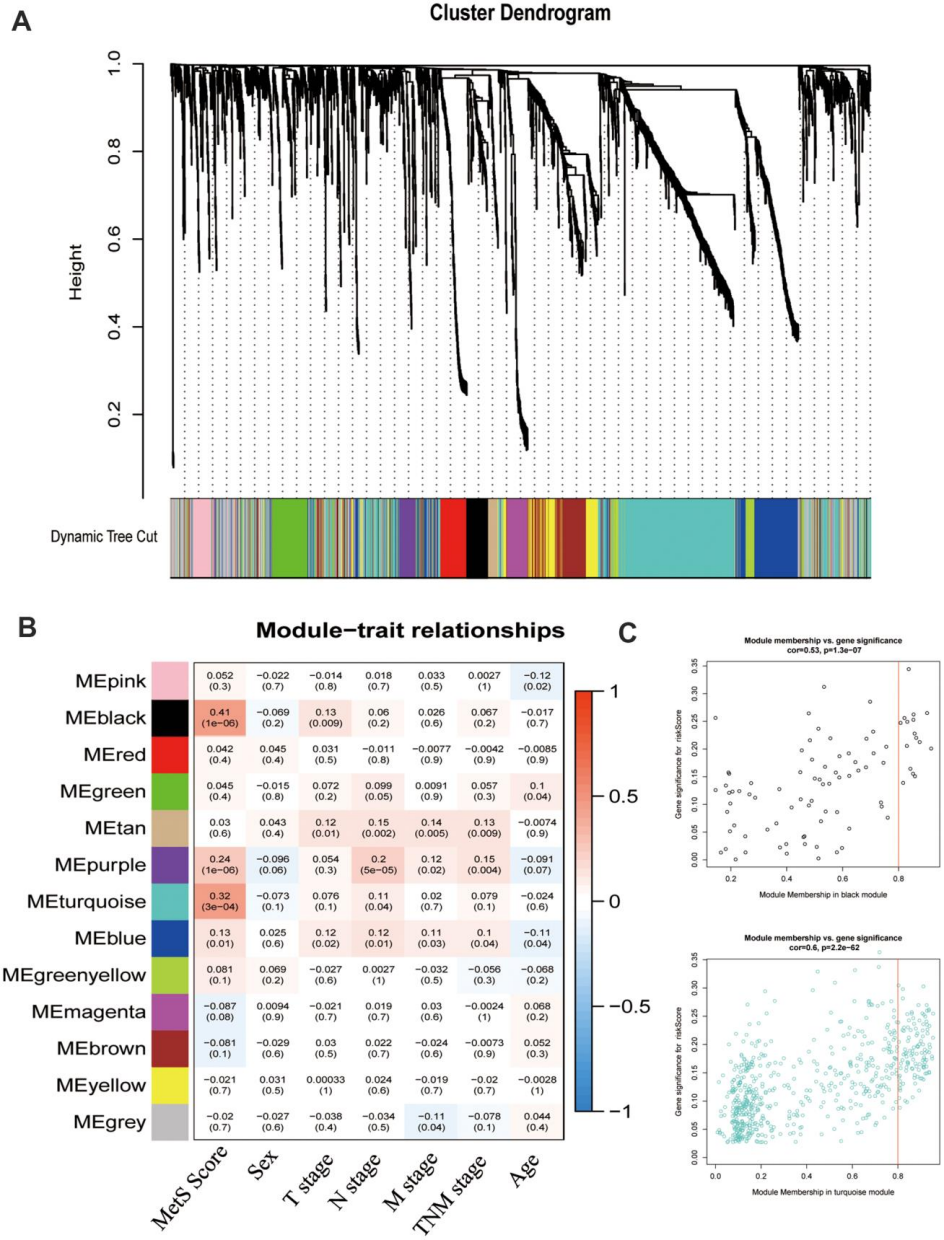
**Weight gene co-expression network analysis (WGCNA) identified metabolic -related modules eigengenes.** (**A**). The clustering dendrograms for the CRC groups. (**B**). Heatmap of module-trait relationships. (**C**). The relationship of module membership and gene expression.

To particularly describe molecular events relative to cancer metabolic progress, the WGCNA was used to identify the module eigengenes that were associated with MetS score. The power (β) of 6 was selected as the soft-power to ensure a scale-free network (scale R2 = 0.95), and ultimately identified 14 co-expressed gene modules (Figure 3A). The correlation between the module eigengenes and the clinical traits were shown in Figure 3B. Particularly, black and turquoise eigengenes were significantly associated with cancer MetS Score (Figure 3C).

### Metabolic signature was associated with drug metabolism

**Figure 4:**
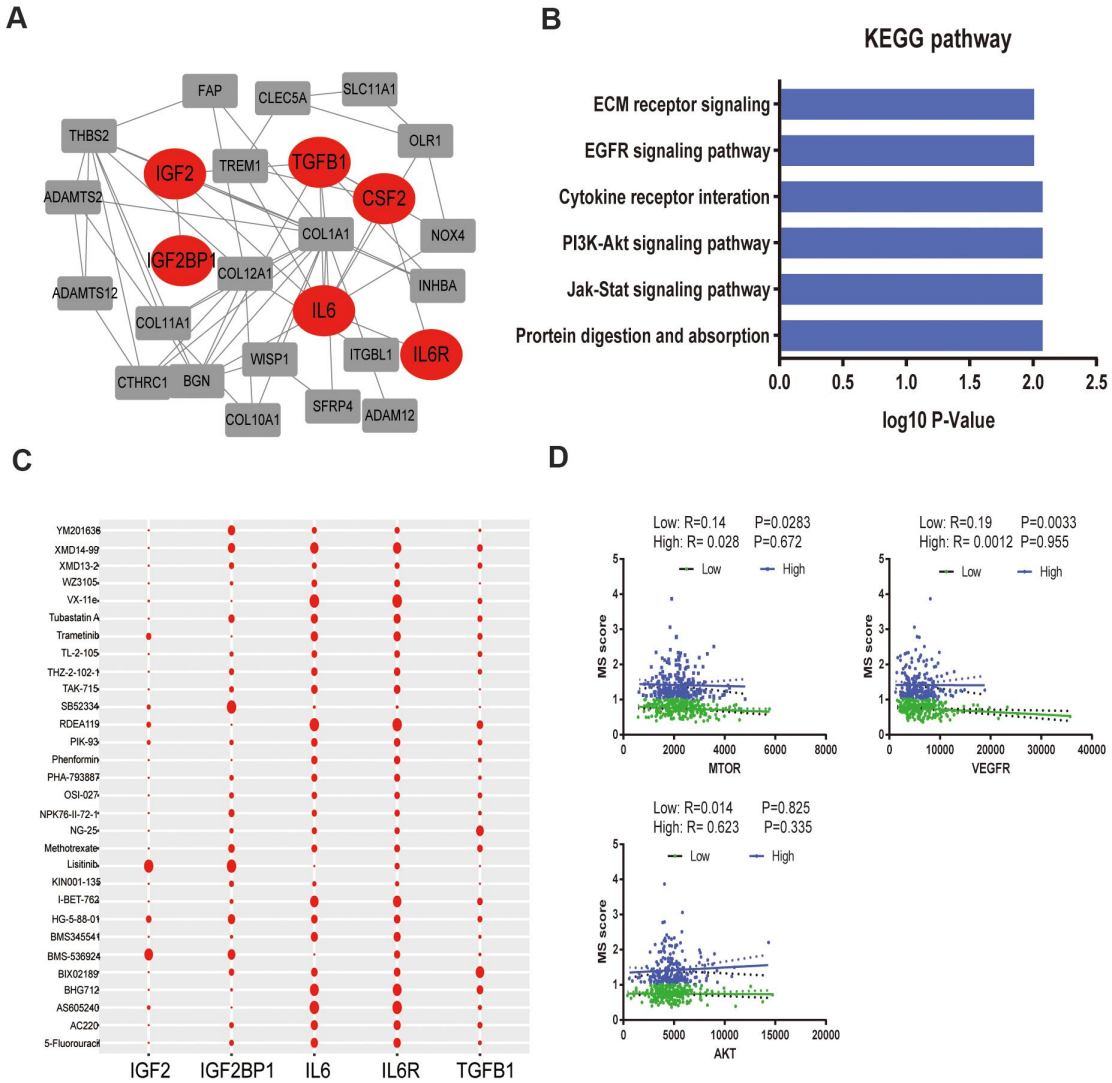
**Functional annotation of black module and drug response.** (**A**). PPI network of black module genes. The known metabolic -related genes were strikingly marked in red; (**B**). Functional annotation for black module; (**C**). Correlation between drugs metabolism and metabolic –related genes, which affect major signaling pathways—IGF, EGFR, mTOR, ERK-MAPK, p53, JNK and p38-MAPK signaling. (**D**). The correlation of gene MetS Score and major drugs metabolism signaling pathways.

To better annotate the black module function, we singled out the 20 central genes in the co-expression network whose MM > 0.8. The known metabolic-related genes were strikingly marked in red (Figure 4A). Functional enrichment showed that these genes owe strong association with classic drug metabolism pathways, such as EGFR signaling, PI3K-mTOR signaling and JAK signaling (Figure 4B). In addition, we compared the gene expression profiles between high- and low- gene MetS score by GSEA. It suggested that the high-MetS score group was closely associated with key drug metabolism process, such as UDP glycosyltransferase, compound transmembrane transporter activity, drug metabolism cytochrome P450 and other enzymes (Supplementary Figure 1). Consistent with black module functional annotation, metabolic syndrome factors (specially for DG, BMI and HDL) also have an immense influence on chemotherapy drug metabolism in our above clinic retrospective investigation (Supplementary Figure 2).

To further detect the effects of metabolic syndrome on drug metabolism, further in-depth study of the correlation of metabolic-related gene expression and chemotherapy drug sensitivity was performed by Pearson correlation, which clarified the pathological mechanisms that link its components with carcinogenesis. We focused on major targeted drug pathways—IGF, EGFR, mTOR, ERK-MAPK, p53, JNK and p38-MAPK signaling. Remarkably, IL-6 and IL6R remarkably improved the effectiveness of targeted therapy administration, yet IGF2 was closely related with drug tolerance (Figure 4C). Specially, low metabolism group was strongly associated with the mTOR pathway and VEGFR pathway without significantly association with AKT pathway (Figure 4D).

### IL6 promoted oncogenic growth in CRC by stimulating mTOR signaling

**Figure 5:**
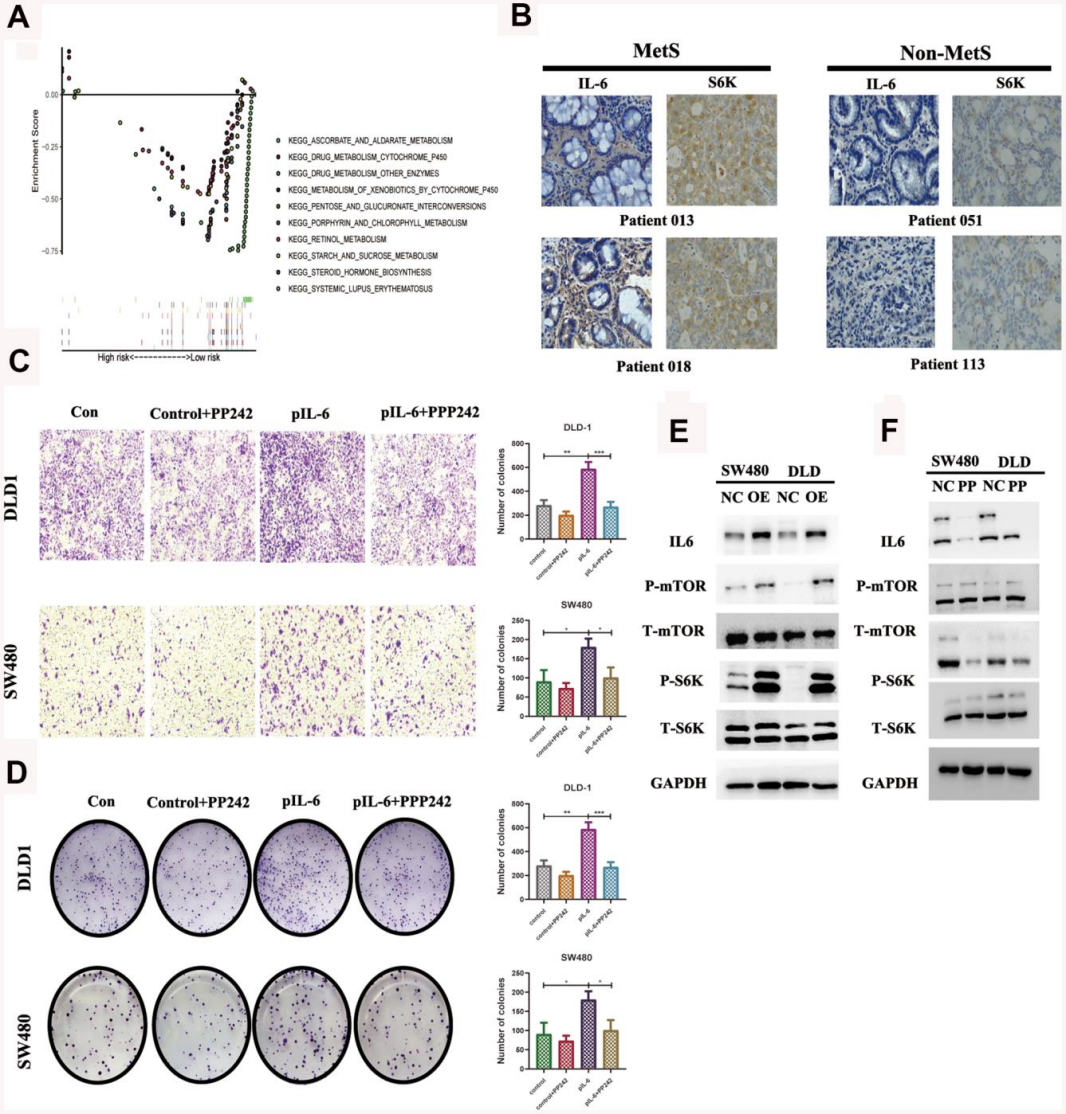
**IL6 promotes oncogenic growth in CRC by stimulating mTOR signaling.** (**A**). GSEA of IL6 in the TCGA CRC cohort; (**B**). Immunohistochemical staining of IL6 in MetS and non-MetS CRC patients; IL6 promotes growth-factor-induced migration (**C**) and invasion of CRC cells (**D**). Cancer cells with or without IL6 overexpression were treated with or without PP242; (**E**) IL6 promoted mTOR signaling in colorectal cancer cells. SW480 and DLD1 expressing ectopic IL6 or vector were analyzed for mTOR signaling by immunoblotting for P-mTOR and P-S6K. GAPDH was used as a loading control; (**F**) Pharmacological inhibition of mTOR signaling by PP242 abrogated IL6 overexpression-induced activation of mTOR signaling. SW480 and DLD1 cells were transfected with IL6 shRNAs or a control shRNA and then treated with PP242. Their effect on mTOR signaling was analyzed by immunoblotting.

We compared the differential expression of key metabolic genes and they all showed high diversity (P< 0.05, Supplementary Figure 3). IL6 was thought to promote tumor growth mainly by paracrine and autocrine methods, which explained the above experimental results to some extent. GSEA revealed that high IL6 expression groups in the TCGA CRC cohort were mainly enriched in KEGG pathways related to drug metabolism, especially the mTOR pathway (Figure 5A). We further specially delineated the protein expression patterns of IL6 and p-S6 by immunohistochemical staining, and they showed higher expression patterns in above MetS CRC patients (Figure 5B). We further detailed investigated the consequence of IL6 overexpression by stably expressing IL6 in SW480 and DLD1. Ectopic expression of IL6 was also sufficient to robustly promote CRC cell migration and transwell compared with control cells (Figure 5C, 5D). On the other hand, IL6 overexpression strongly potentiates the activity of mTOR pathway as shown by much enhanced S6K1 and mTOR phosphorylation (Figure 5E). Given the previous research, we speculated that whether IL6 participated in the regulation of the activity of PP242 activity, a mTOR kinase inhibition. As shown in Figure 5F, IL6 ectopic expression enhanced PP242 drug susceptibility in colorectal cancer cells, which completely abolished the activity of mTOR pathway. We also found that PP242 strongly inhibited the invasion of IL6-overexpressing CRC cells (Figure 5C, 5D). The above results suggested that overexpression of IL6 prompted colorectal cancer highly dependent on mTOR-S6K signaling and more sensitive to mTOR kinase inhibitors.

## DISCUSSION

MetS and its related complications are serious health problems, and the global prevalence has exceeded 23.7%, without significant statistic evidence in gender. Emerging evidences have demonstrated that MetS is a vital factor for the development and malignant progression of various cancers [11]. Patients with MetS have a higher risk for an increased in the 30-day postoperation mortality rate, postoperative complications, and recurrence of colorectal adenoma [12]. Therefore, exploring the relationship between MetS and CRC becomes extremely important.

The results of the present study suggested that the components of MetS were closely related with poor prognosis of CRC. Based on the results of univariate Cox analysis, we found that patients with hypertension, with low HDL cholesterol, who were underweight or who had diabetes/hyperglycemia were more likely to have poor survival (Figure 1A–1E). In addition, we constructed a clinical nomogram based on the MetS syndrome and other clinical items, followed by analyzing the relationship between different subgroups and their prognosis. A higher nomogram score was associated with a poorer prognosis of patients with CRC. It is promising to utilize this nomogram in the future to predict the prognosis of CRC patients (Figure 1F, 1G). Although the impact of the components of MetS on the clinical prognosis of CRC patients has been confirmed, the underlying mechanism remains unclear. The possible association mechanism between these two risk factors was firstly described in this paper and focused on shedding light on the candidate signature genes and biological events occurring during CRC progression. We identified 11 key metabolic genes from recognized literature, including TGFB, IGF2BP1, IGFL1, IGF2BP3, CHS2, IGF1R, IL6, IL6R, CSF1, CSF3 and IGF2. CRC patients were divided into high- and low-risk groups based on the MetS-gene score. The prognosis of the low-risk group was significantly better than the high-risk group (Figure 2). Using WGCNA, we found that MetS was also related to drug metabolizing enzymes (DMEs) and pathways (Figure 3). Comparing the high- and low-MetS groups with multiple pathways, MetS-gene score was proven strongly associated with the mTOR pathway and VEGFR pathway. Moreover, IL-6 was also found to be highly associated with the drug sensitivity and resistance of mTOR inhibitors in CRC patients. We speculate that drug resistance is a crucial cause contributing to the poor prognosis of CRC caused by MetS, and inhibiting the expression of IL-6 can increase drug susceptibility.

Drug metabolizing enzymes (DMEs), including uridine diphospho-glucuronosyltransferases (UGTs), glutathione-S-transferases (GSTs), and cytochromes, can degrade molecular drugs and convert some antitumor drugs into inactive metabolites, causing resistance against chemotherapeutic agents and drug inefficiency [12, 13]. Thus, less sensitivity or efficiency of antitumor drugs (e.g., irinotecan, rapamycin, 5-FU, cisplatin) are owed in treating the corresponding tumors on account of the metabolism of DMEs [14]. mTOR-S6k is one of the most common deregulated pathways in various cancers, and overactivation of the mTOR pathway is closely related to cell growth, metabolism, aging, insulin resistance and obesity, of which are the most common risk factors resulting in MetS [15, 16]. Based on these findings, rapamycin, as an allosteric inhibitor of mTOR, was approved for the treatment of various cancers by forming a complex with FKBP12 to inhibit mTORC1 activity [17]. However, many cancers, including CRC, demonstrated resistance to the antitumor effects of rapamycin [18, 19]. In our study, we found that IL-6 could promote the malignant biological properties of CRC via mTOR-S6K signaling, which provided a good therapeutic suggestion for clinical practice. CRC patients with high expression of IL-6 would be more applicable to inhibitors of the mTOR pathway.

IL-6, a pleiotropic cytokine, can induce a chronic state of low-degree inflammation and insulin resistance, which is also positively related to CRC tumorigenesis. Excessive fat tissue in obese people can secrete more IL-6, adiponectin and leptin to promote metabolic homeostasis [20]. EGFR-induced mTOR can stimulate the expression of IL6 through the classic pathway and reprogram IL-6 nonresponsive cells into IL-6 responder cells, to further affect the sensitivity of the tumor cells to IL-6 [21]. The results of the combined analysis of IL-6 and tyrosine kinase inhibitors (TKI) showed that high levels of IL-6 can upregulate the mTOR signaling pathway and induce tumor cell resistance to TKI therapy [13]. Inhibiting the expression of IL-6 or blocking the IL-6 pathway would alleviate tumor cell resistance to TKI therapy and enhance the antitumor efficiency of TKI, which was consistent with the founding of our study. However, the precise mechanism behind this enhancement is still unknown and further study is needed.

IL-6 was also involved in other drug-metabolizing enzymes and anticancer pathway activities. IL-6 can manipulate the expression of CYP2E1 and CYP1B1 to induce tumorigenesis by activating carcinogens and causing DNA damage through the JAK/STAT and PI3K/AKT pathways [22]. The STAT-3 signaling pathway was also found to play a key role in meditating the effect of IL-6 on promoting tumor progression and treatment resistance by inhibiting cancer cell apoptosis and stimulating tumor-associated factors among various signaling pathways [20, 23]. IL-6 can inhibit the chemotherapeutic efficacy of 5-FU, the most commonly used chemotherapy for CRC, by activating the IL-6R/GP130 signaling pathway and the levels of P-AKT, P-ERK and P-STAT3 [24]. The use of the antihuman IL-6 receptor monoclonal antibody can weaken the tumor phenotype and 5-FU resistance [25]. In conclusion, IL-6 mediates a series of reactions in the development of CRC and drug resistance.

In our study, another gene that attracts our attention most is IGF2, which was found to be closely associated with drug tolerance in CRC. IGF2 has been found to be a mitogen that is mainly expressed in the cell cytoplasm and vesicles, and significantly drives cell proliferation and promotes tumorigenesis [26, 27]. Insulin/IGF system can regulate cell proliferation, differentiation, apoptosis, glucose transport and energy metabolism [28]. Insulin/IGF system is also a decisive factor in the development of CRC malignancy and metastasis and promised to be a therapeutic target of this disease [29]. Some researches hold that IGF2 participated in the regulation of the cancer cell secretion of VEGF and further impacted the power of VEGFA antibody [30]. In the study of CRC for cetuximab efficacy, EGFR inhibition was found in a part of patients with IGF2 overexpression, which could also weaken the efficacy of cetuximab in functional studies [31, 32]. IGF2 has been proven to function through the PI3K-AKT-mTOR signaling pathway [33]. Using mTOR inhibitors could weaken the promoting effect of IGF on the proliferation and viability of tumor cells, while combining IGF2 inhibitors could provide a better curative effect on patients with adrenocortical carcinoma [34]. Therefore, anti-IGF2 treatments are very promising for reversing drug resistance and inhibiting tumorigenicity.

The strengths of this research include exploring and verifying the relationship between MetS and the prognosis and survival of CRC from the perspective of epidemiology and molecular mechanisms. We further explored the possible mechanism by which MetS leads to the poor prognosis of CRC, and partially verified it with *in vitro* experiments. Nevertheless, there were still several limitations. First of all, the sample size was small in some of the analyses, which may have limited the statistical accuracy. Secondly, this article was a monocenter study; therefore, to avoid bias and deviations, our findings should be validated using multicenter prospective studies. Thirdly, it was difficult to estimate the effect of MetS treatment on the prognosis of CRC patients, for whom comprehensive data on MetS treatment could be obtained, which may confuse the causal relationship between MetS and CRC prognosis. Fourth, some confounding factors were not assessed in this study, such as the history of smoking, drinking, occupation, and other cancers.

In conclusion, the current study reveals that MetS is a risk factor for a poor CRC prognosis and promises to be a potential prognostic indicator. IL-6 and IGF2 affect mainly the chemosensitivity and drug resistance of tumors through the mTOR pathway, resulting in a poor CRC prognosis for patients with MetS. Further study demonstrated that the combined treatment of inhibition of IL6 and mTOR pathway is expected to become a new treatment for CRC patients with MetS, which will be verified in future experiments.

## MATERIALS AND METHODS

### Patients and clinical outcome assessment

This study utilized data from the First and the Second Affiliated Hospital of Wenzhou Medical University and public databases. We performed a retrospective study of colorectal cancer patients in these two institutions in Wenzhou, China (Wenzhou Medical University), from January 2010 to January 2016. The inclusion criteria were as follows: 1. histopathological diagnosis of colorectal cancer; 2. all data for patients diagnosed with colorectal cancer for the first time without any treatment; 3. complete pathology, laboratory, and follow-up data. Patients with unknown included variables were excluded. The following demographic, clinical, and pathology data were used: T stage, N stage, M stage, pathological stage, tumor history, laboratory test results (age, sex, body-mass index (BMI), TG, HDL-C, CEA, creatinine). Pathologists assessed the tumor stage according to the 7th edition of the AJCC TNM staging guidelines. Patients who received only neoadjuvant chemotherapy or surgery or those with unknown included variables were excluded. Metabolic syndrome was internationally defined as included more than three criteria: 1) BMI greater than or equal to 25.0 kg/m^2^; 2) diagnosed with diabetes; 3) diagnosed with hypertension, SBP/DBP >140/90 mmHg; 4) blood HDL-C < 0.9 mmol/L, 5) blood TG > 1.7 mmol/L. Totally, there were 716 eligible cases selected in our study, 15.1% were diagnosed with MetS. All of these patients were followed up, and recurrent and dead patients were recorded during the follow-up. The time was cut off in March 2020. The study protocols were approved by the Wenzhou Medical University Ethics Committee. All procedures adhere to the BRISQ Guidelines for reporting research on human biospecimens.

**Figure 6:**
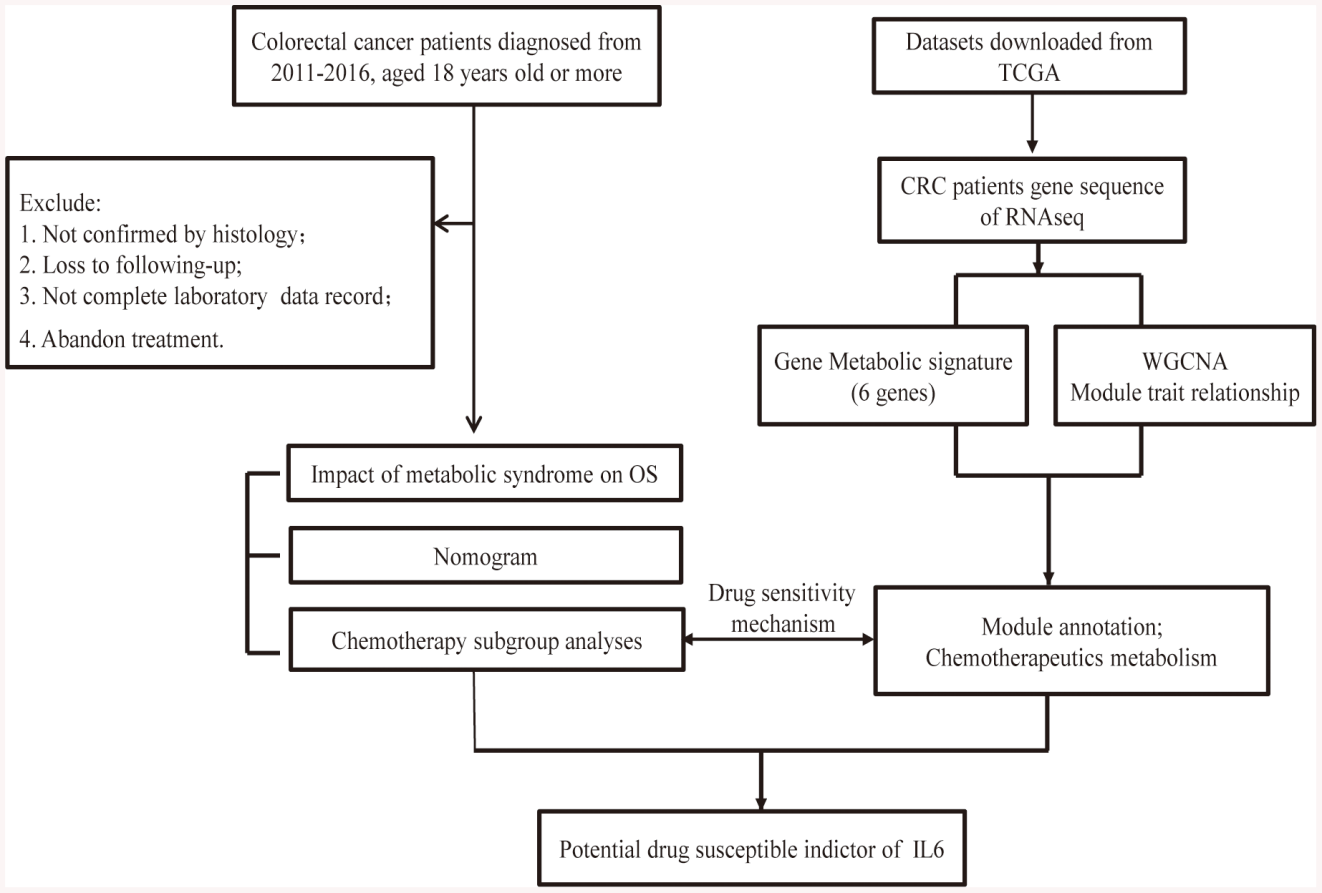
**Flow chart of the experimental design and main process.**

We also retrospectively selected colorectal cancer gene expression and relative clinicopathological data from the TCGA database (https://portal.gdc.cancer.gov/). Raw microarray data Affymetrix were downloaded and normalized using the limma package. The detailed working algorithm was demonstrated in the Figure 6.

### Construction nomogram models

An OS nomogram was constructed based on the prognostic factors derived from multivariate Cox regression analysis to predict 1-, 3- and 5-year survival rate. Each patient could sum up corresponding variable score and finally establish predictive measures of OS. The nomogram was generated using ggplot packages together with R software. The calibration curve for predicting 1-, 3- and 5-year OS indicated that the nomogram-predicted survival was closely corresponded with actual survival outcomes. The survival analysis was conducted using rms, survivalROC, survcomp and survival package. Hazard ratios (HRs) and 95% confidence intervals (CIs) were recorded.

### Evaluation of TCGA gene metabolic score

R language survival package was used to perform the Cox regression model analysis. The key metabolic gene lists were obtained from literature investigation the molecular mechanism of MetS from Pubmed [10, 35], and then applied to multivariate Cox regression analysis. The MetS score of each patient was calculated with the expression level and its relative coefficient. On the basis of the median score as the cut-off setline, these patients were divided into high- and low-MetS subgroups. Log-rank test was performed to calculate the corresponding hazard ratios (HRs) and 95% confidence interval (CI).

### Weighted gene co-expression network analysis

The weighted gene co-expression network analysis (WGCNA) hierarchically clustered module eigengenes of the clinical features based on co-expression relationships, thoroughly explored the biological processes and molecular mechanisms behind cancer metabolic disorders [36]. In this study, we constructed WGCNA to explore the colorectal cancer dataset to identify gene modules associated with expression patterns of MetS score and pathology factors. The co-expression network was constructed by the R package WGCNA. The connectivity degree of each node of the network was calculated by STRING database and reconstructed via Cytoscape software.

### Functional annotation

The signaling pathway and molecular function underlying MetS score were explored with GSEA (Version 4.0.1). The number of permutations was set at 1000, and P < 0.05 and an FDR < 0.25 were considered statistically significant. Gene ontology (GO) enrichment analysis was performed with the DAVID platform.

### GDSC (Genomics of drug sensitivity in cancer)

GDSC, a large-scale drug screening data screened on a panel of 990 human cancer cell lines, concentrates on providing publicly available tumor treatment genome data and identifying potential tumor treatment targets. (http://www.cancerrxgene.org/gdsc1000/) [37–39]. Gene mutations of the cancer genomes greatly affect clinical outcomes and drug targets respond. To analyze the correlation of expression and drug sensitivity, the Pearson correlation coefficient of transcript levels was calculated.

### Immunohistochemistry

Above-mentioned 20 CRC specimens were collected, including 10 MetS and 10 non-MetS CRC tissues. Two researchers evaluated the staining results independently and scored the staining intensity of immunostaining as: 0 (negative), 1 (weakly positive), 2 (moderately positive) and 3 (strongly positive). The primary antibody against P-S6 and IL6 was used at a concentration of 1:200. For quantitative analysis, a Histo (H) score was calculated based on the staining intensity and percentage of stained cells using Aperio ScanScope system (Vista, CA, USA).

### Colony formation and transwell migration assay

A number of 1 × 10^3^ DLD1 and SW480 were inoculated in six-well plates and incubated at 37° C for 5-7 days. Then, we treated the colonies with PP242 or vector for 5 days. Colonies were fixed with 4% paraformaldehyde formaldehyde (Solarbio, Beijing, China) followed by staining with crystal violet (Sigma-Aldrich). The number of colonies with more than 50 cells was calculated. Transwell migration experiments were used to confirm the migration ability of DLD1 and SW480, and 5 × 10^4^ cells were added to the upper chamber placed in a 24-well plate, with serum-free medium. Meanwhile, medium containing 15% serum was added to the lower chamber. Taking cell images at 100× magnification 5 random microscopic fields of view were selected to count the number of migrated cells.

### Antibodies and western blot analysis

An equal amount of protein was subjected to SDS-PAGE. Proteins were transferred into PVDF membranes, and the blots were incubated with the following different primary antibodies: Rabbit mTOR, p-mTOR (S2448), S6K, p-S6K (T389), p-S6 from Cell Signaling Technology, and IL6 from Proteintech. HRP-labeled GAPDH, anti-mouse and anti-rabbit antibodies were purchased from Santa-Cruz Biotechnology. All primary antibodies were confirmed to be reactive only to the targets by the manufacturer and used at 1:1000, and secondary antibodies were used at 1:5000.

### Statistical analysis

R software and Stats were used for statistical analyses. Continuous variables were exhibited for means, medians, range, and standard deviation (SD) and compared using an independent t test or Wilcoxon test; Spearmen correlation coefficient was used for variable correlation; Chi-square test was used to analyze categorical variables. The log-rank survival test and the results were shown in the forest plot. All statistical tests were two-sided and P <0.05 was considered statistically significant.

### Date accessibility

The data are available in the TCGA datasets.

## Supplementary Material

Supplementary Figures

Supplementary Table 1
