# Supplementary material for: Clinical and genomic characteristics of metabolic syndrome in colorectal cancer
Source: Aging (Albany NY). 2021 Feb 11;13(4):5442–60. doi: 10.18632/aging.202474 (PMC7950286; doi:10.18632/aging.202474)
Supplement: Supplementary Figures [file aging-13-202474-s001.pdf]

SUPPLEMENTARY FIGURES

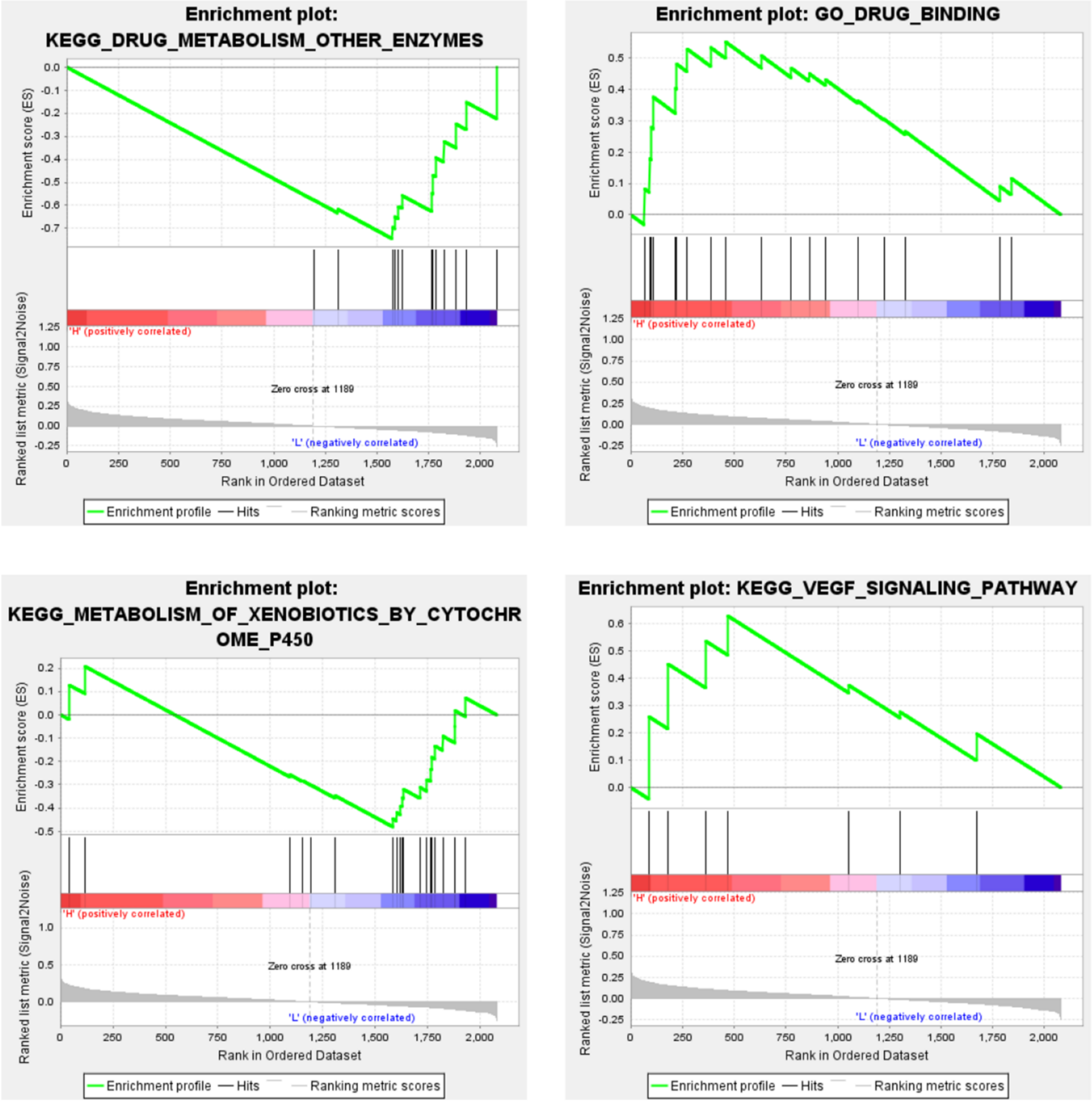

Supplementary Figure 1. GSEA of MetS Score. High-MetS Score group was closely associated with drug metabolism pathway.

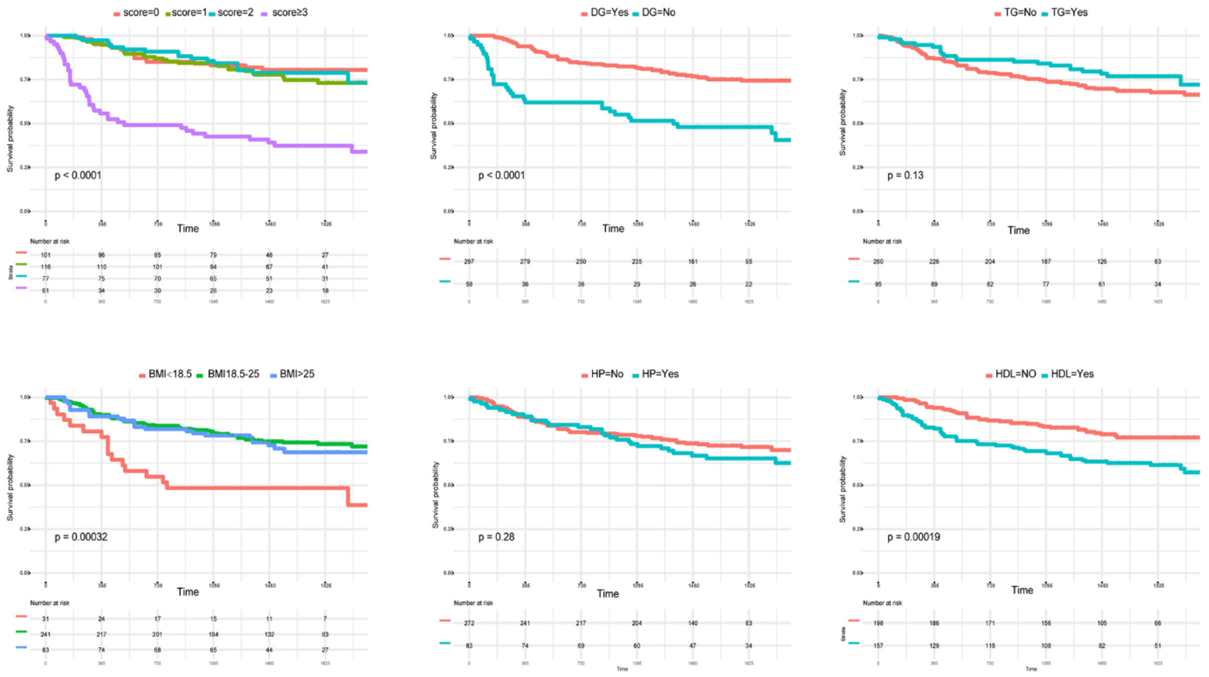

Supplementary Figure 2. The effect of metabolic variables on survival in CRC chemotherapy subgroups. The effects of MetS, diabetes, obesity, dyslipidemia weight and hypertension on survival in CRC chemotherapy subgroups.

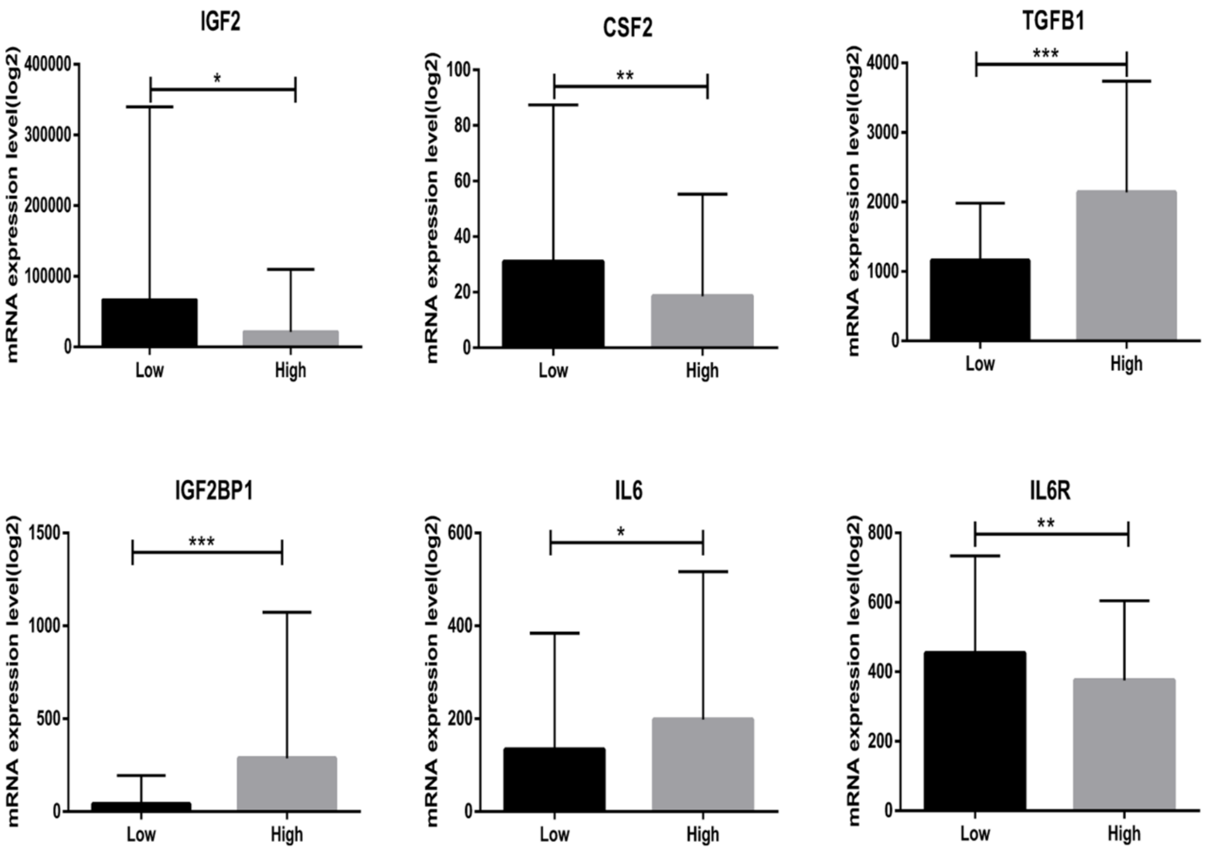

Supplementary Figure 3. The metabolic –related gene expression in low- and high- MetS score group.
