# Supplementary material for: Clinical and genomic characteristics of metabolic syndrome in colorectal cancer
Source: Aging (Albany NY). 2021 Feb 11;13(4):5442–60. doi: 10.18632/aging.202474 (PMC7950286; doi:10.18632/aging.202474)
Supplement: Supplementary Table 1 [file aging-13-202474-s002.pdf]

SUPPLEMENTARY TABLE

Supplementary Table 1. Baseline clinicopathological parameters of CRC patients.

Characteristics	Number(%)
Age (range)	
Median (range)	24-85
< 60	281
≥60	433
Gender	
Male	427
Female	289
Chemotherapy	
No	361
Yes	355
CEA	
<5	426
≥5	290
T stage	
T1	34
T2	68
T3	84
T4	531
N stage	
N0	369
N1	181
N2	166
M stage	
M0	601
M1	115
Pathological Stage	
1	87
2	260
3	252
4	118
BMI	
≤18.5	75
18.5-25	494
≥25	147
Hypertension	
No	519
Yes	197
Diabetes	
No	630
Yes	86
HDL	
<0.9	365
≥0.9	349
TG	
<1.7	521
≥1.7	193
Renal failure	
No	593
Yes	123
MetS	
0	187
1	247
2	174
3-5	108
